# Supplementary material for: Convergent High O2 Affinity but Distinct ATP-Mediated Allosteric Regulation of Hemoglobins in Oviparous and Viviparous Eremias Lizards from the Qinghai-Tibet Plateau
Source: Animals (Basel). 2024 May 11;14(10):1440. doi: 10.3390/ani14101440 (PMC11117339; doi:10.3390/ani14101440)
Supplement: Supplementary file 1 [file animals-14-01440-s001.zip › animals-2962560-supplementary.pdf]

Table S1. PCR primers are used to amplify adult-expressed *α*- and *β*-type globin genes of high- and low-altitude *E. argus* and *E. multiocellata.*

Genes	Primer names	Sequence (5’ to 3’)
αA	lbmx-A-F	GACGGCACCCGAGACTTTG
αA	lbmx-A-R	TCAAGAGCAGGTTTATTGTGGG
αD	lbmx-D-F-1	CTGCCTGCTGCCAAGATG
αD	lbmx-D-R-1	GCGGAGTGCCCGAGAC
αD	mdmx-D-F-2	GCCACCCATCGAGCAGTT
αD	mdmx-D-R-2	GCGGCTCATCGGTACTTCT
β1	lbmx-B1-F	CTCCAGCTCAGCCCAGAA
β1	lbmx-B1-R	GAGACCAGCAGGAGGCG
β2	lbmx-HbB2-F	CCCAGAAGGTTTCGTCCAG
β2	lbmx-HbB2-R	TTTATTAGCTAGTGGCCCCG

Table S2a. List of α-globin amino acid sequences included in the phylogenetic analyses.

Species	globin	Accession	Source	Family
Human (*Homo sapiens*)	αA	P69905	Uniprot	Hominidae
	αD	Q6B0K9	Uniprot	
	αE	P02008	Uniprot	
Chicken (*Gallus gallus*)	αA	P01994	Uniprot	Phasianidae
	αD	P02001	Uniprot	
	αE	P02007	Uniprot	
Anole (*Anolis carolinensis*)	αA		ref1	Dactyloidae
	αD		ref1	
Gekko japonicus	αA	XP_015265387.1	Genebank	Gekkonidae
Iguana iguana	αA	P18974	Uniprot	Iguanidae
Varanus komodoensis	αA	KAF7241950.1	Genebank	Varanidae
	αD	KAF7243096.1	Genebank	
Pogona vitticeps	αA	annotated from genome	ref2	Agamidae
	αD	XP_020667603.1	Genebank	
Phrynocephalus erythrurus	αA		ref3	Agamidae
	αD		ref3	
Phrynocephalus przewalskii	αA		ref3	Agamidae
	αD		ref3	
Podarcis muralis	αA	XP_028561926.1	Genebank	Lacertidae
	αD	XP_028562646.1	Genebank	
Zootoca vivipara	αA	XP_034986452.1	Genebank	Lacertidae
	αD	XP_034986451.1	Genebank	
Lacerta agilis	αA	XP_033023270.1	Genebank	Lacertidae
	αD	XP_033023740.1	Genebank	
Sceloporus undulatus	αA	XP_042294013.1	Genebank	Phrynosomatidae
	αD	XP_042294444.1	Genebank	

Table S2b. List of β-globin amino acid sequences included in the phylogenetic analyses.

Species	globin	Accession	Source	Family
Human (*Homo sapiens*)	β	P68871	Uniprot	Hominidae
Chicken (*Gallus gallus*)	β	P02112	Uniprot	Phasianidae
Anolis carolinensis	β1	XP_003229662.1	Genebank	Dactyloidae
	β2	named as β^{I} in ref1	ref1	
Gekko japonicus	β2	XP_015266989.1	Genebank	Gekkonidae
Iguana iguana	β1	P18987	Uniprot	Iguanidae
	β2	P86390	Uniprot	
Amblyrhynchus cristatus	β2	P86391	Uniprot	Iguanidae
Varanus komodoensis	β	KAF7236611.1	Genebank	Varanidae
Varanus albigularis	β1	P18993	Uniprot	Varanidae
Pogona vitticeps	β1	XP_020647556.1	Genebank	Agamidae
Phrynocephalus erythrurus	β1		ref3	Agamidae
	β2		ref3	
Phrynocephalus przewalskii	β1		ref3	Agamidae
	β2		ref3	
Podarcis muralis	β1	XP_028581395.1	Genebank	Lacertidae
	β2	XP_028581397.1	Genebank	
Zootoca vivipara	β1	XP_034972328.1	Genebank	Lacertidae
	e	XP_034970899.1	Genebank	
Lacerta agilis	β1	XP_033001617.1 (annotated as β2-like)	Genebank	Lacertidae
	β2	XP_033001613.1 (annotated as β1-like)	Genebank	
Sceloporus undulatus	β1	XP_042316261.1	Genebank	Phrynosomatidae
	β2	XP_042316446.1	Genebank	

The name of β1 and β2 in anole were inaccurate in the study of Storz JF et al. (2011). We aligned the β^{II} in their study to the nr database with the blastp of NCBI and found that β^{II} was completely consistent with the β1 annotated in the anole genome (ref4). The re-annotation of the anole genome by Lu S (2017) (ref3) also confirmed that β^{I} and β^{II} in the study of Storz JF et al. (2011) should be β2 and β1. In addition, the β1 and β2 of *L. agilis* also have the same naming problem as anole. Blastp results found that its β1 is most similar to β2 of *P. muralis*, while its β2 is most similar to β1 of *Z. vivipara* and *P. muralis*. Therefore, in this study, we renamed the β1 and β2 of the anole and *L. agilis* based on the blastp results.

ref1: Storz JF et al. (2011). Developmental regulation of hemoglobin synthesis in the green anole lizard Anolis carolinensis. *The Journal of Experimental Biology* **214**, 575-81.

ref2: Georges A et al. (2015). High-coverage sequencing and annotated assembly of the genome of the Australian dragon lizard *Pogona vitticeps*. *Gigascience* **4**, s13742-015-0085-2.
ref3: Lu S (2017). The adaptive mechanism of globin family to high altitude hypoxia in *Phrynocephalus* lizards (Doctor's thesis in Chinese).

Table S3. The theoretical molecular weight (MW) of α- and β-type globins sequenced in high- and low-altitude *E. argus* and *E. multiocellata*

MW	αA	αD1	αD2	β1-1	β1-2	β2
E. argus-H	14903.14	15990.36	-	16182.82	16198.82	16026.47
E. argus L	14903.14	15972.32	-	16182.82	16198.82	16045.53
E. multiocellata-H	14903.14	15999.37	16109.52	16254.97	-	16026.47
E. multiocellata-L	14903.14	15999.37	16109.52	16285.98	-	15948.4

Table S4. O_2 affinities (indexed as P_{50} values) and Hill's cooperativity coefficients (indexed as n_{50} values) for high- and low-altitude *E. argus* and Hbs measured in pH 7.8, 7.4 Hepes buffers at 25 °C and 37 °C in the absence (stripped) and presence of Cl^- (added as 0.1 mol/L KCl) and/or ATP (100-fold molar excess over tetrameric Hbs).

Means ± SEM	*E. argus*-High				*E. argus*-Low			
Temperature	25 °C		37 °C		25 °C		37 °C	
pH	7.8	7.4	7.8	7.4	7.8	7.4	7.8	7.4
P_{50} (mmHg)								
Stripped	2.21±0.03[a]	2.77±0.03[a]	4.02±0.03[a]	4.54±0.10[a]	3.22±0.02[a]	3.87±0.01[a]	5.04±0.08[a]	5.44±0.08[a]
+KCl	3.49±0.02[b]	4.06±0.05[b]	4.83±0.05[b]	5.71±0.08[b]	4.38±0.02[b]	5.01±0.08[b]	5.64±0.03[b]	6.57±0.13[b]
+ATP	7.54±0.05[c]	11.50±0.07[c]	11.00±0.07[c]	14.48±0.10[c]	8.51±0.06[c]	12.51±0.05[c]	11.95±0.06[c]	15.70±0.07[c]
+KCl +ATP	7.36±0.06[d]	11.16±0.11[c]	11.23±0.03[c]	14.84±0.11[d]	8.31±0.06[c]	12.10±0.08[d]	12.14±0.03[c]	16.06±0.05[d]
n_{50}								
Stripped	2.54±0.05	2.39±.11	1.89±0.05	2.01±0.05	2.48±0.04	2.46±0.08	2.07±0.05	2.05±0.03
+KCl	3.08±0.11	2.89±0.06	2.29±0.06	2.38±0.02	2.48±0.07	2.78±0.06	2.13±0.02	2.07±0.03
+ATP	2.46±0.03	2.65±0.03	2.52±0.04	2.52±0.06	2.37±0.05	2.59±0.04	2.36±0.03	2.34±0.04
+KCl +ATP	2.68±0.06	2.69±0.04	2.53±0.04	2.49±0.02	2.42±0.08	2.41±0.03	2.45±0.03	2.39±0.03

Different letters of right superscript on P_{50} values in each population Hbs indicate the significant differences between the stripped state and conditions for adding KCl and/or ATP under the same temperature and pH condition according to the one−way ANOVA using the LSD (equal variances) and Games-Howell (unequal variance) method of post hoc multiple comparisons.

Table S5. O_2 affinities (indexed as P_{50} values) and Hill's cooperativity coefficients (indexed as n_{50} values) for high- and low-altitude *E. multiocellata* Hbs measured in pH 7.8, 7.4 Hepes buffers at 25 °C and 37 °C in the absence (stripped) and presence of Cl^- (added as 0.1 mol/L KCl) and/or ATP (100-fold molar excess over tetrameric Hbs).

Means ± SEM	*E. multiocellata*-High				*E. multiocellata*-Low			
Temperature	25 °C		37 °C		25 °C		37 °C	
pH	7.8	7.4	7.8	7.4	7.8	7.4	7.8	7.4
P_{50} (mmHg)								
Stripped	2.62±0.02^a	3.25±0.03^a	4.17±0.03^a	4.89±0.05^a	3.26±0.02^a	4.05±0.03^a	4.69±0.02^a	5.46±0.04^a
+KCl	3.28±0.03^b	3.99±0.05^b	4.77±0.03^b	5.38±0.03^b	4.20±0.02^b	4.98±0.03^b	5.71±0.02^b	6.35±0.04^b
+ATP	5.34±0.01^c	7.14±0.02^c	7.96±0.06^c	10.43±0.08^c	6.47±0.05^c	8.34±0.05^c	9.65±0.01^c	11.52±0.06^c
+KCl +ATP	6.38±0.04^d	8.16±0.02^d	8.99±0.06^d	10.59±0.11^c	7.46±0.04^d	9.48±0.05^d	10.68±0.04^d	12.67±0.10^d
n_{50}								
Stripped	2.19±0.07	2.42±0.07	2.18±0.02	2.26±0.09	2.40±0.06	2.55±0.11	2.12±0.01	2.13±0.05
+KCl	2.47±0.07	2.55±0.06	2.42±0.06	2.44±0.06	2.82±0.08	2.98±0.07	2.30±0.04	2.30±0.04
+ATP	2.58±0.04	2.93±0.07	2.41±0.05	2.63±0.07	2.39±0.06	3.01±0.04	2.41±0.04	2.52±0.03
+KCl +ATP	2.85±0.12	2.82±0.04	2.68±0.05	2.85±0.05	2.25±0.03	2.58±0.03	2.44±0.04	2.45±0.06

Different letters of right superscript on P_{50} values in each population Hbs indicate the significant differences between the stripped state and conditions for adding KCl and/or ATP under the same temperature and pH condition according to one−way ANOVA using the LSD (equal variances) and Games-Howell (unequal variance) method of post hoc multiple comparisons ($P < 0.05$).

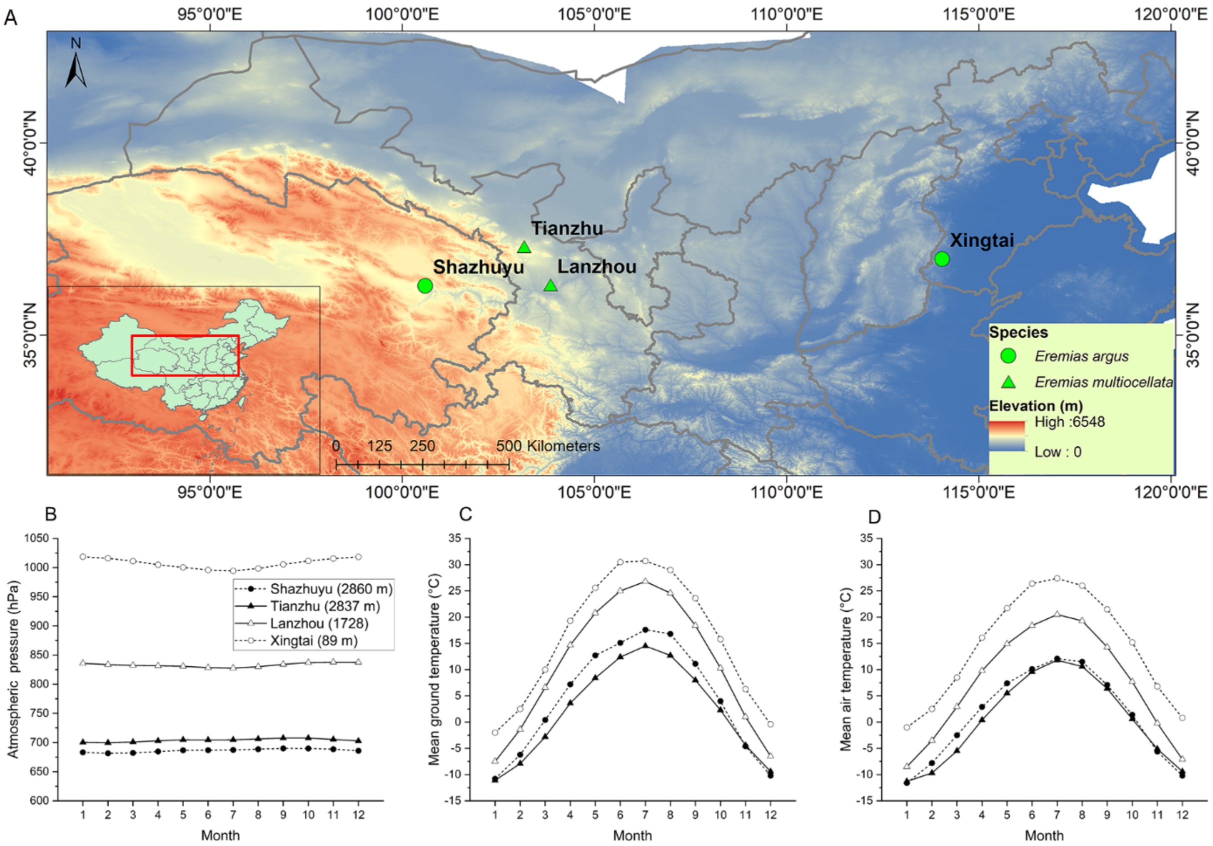

Figure S1 The geographical location (A) and climate data (B, C, D) from 1981 to 2010 of four sample sites of *Eremias argus* (Shazhuyu and Xingtai) and *Eremias multiocellata* (Tianzhu and Lanzhou). B, atmospheric pressure; C, mean ground temperature; D, mean air temperature.

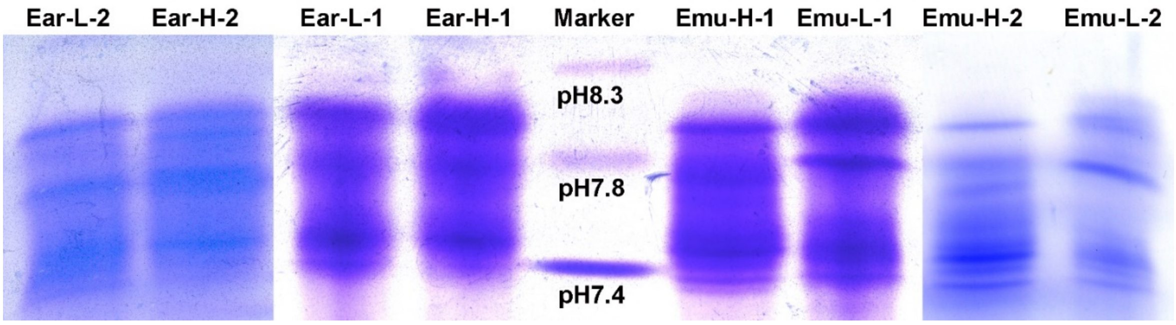

Figure S2 Isoelectric focusing (pH 3–10) of the pooled purified isoform Hb mixture from high-altitude (Ear-H) and low-altitude (Ear-L) *E. argus*, and high-altitude (Emu-H) and low-altitude (Emu-L) *E. multiocellata*. Samples in lanes Ear-H-2, Ear-L-2, Emu-H-2 and Emu-L-2 are dilutions of samples in lanes Ear-H-1, Ear-L-1, Emu-H-1 and Emu-L-1, respectively.

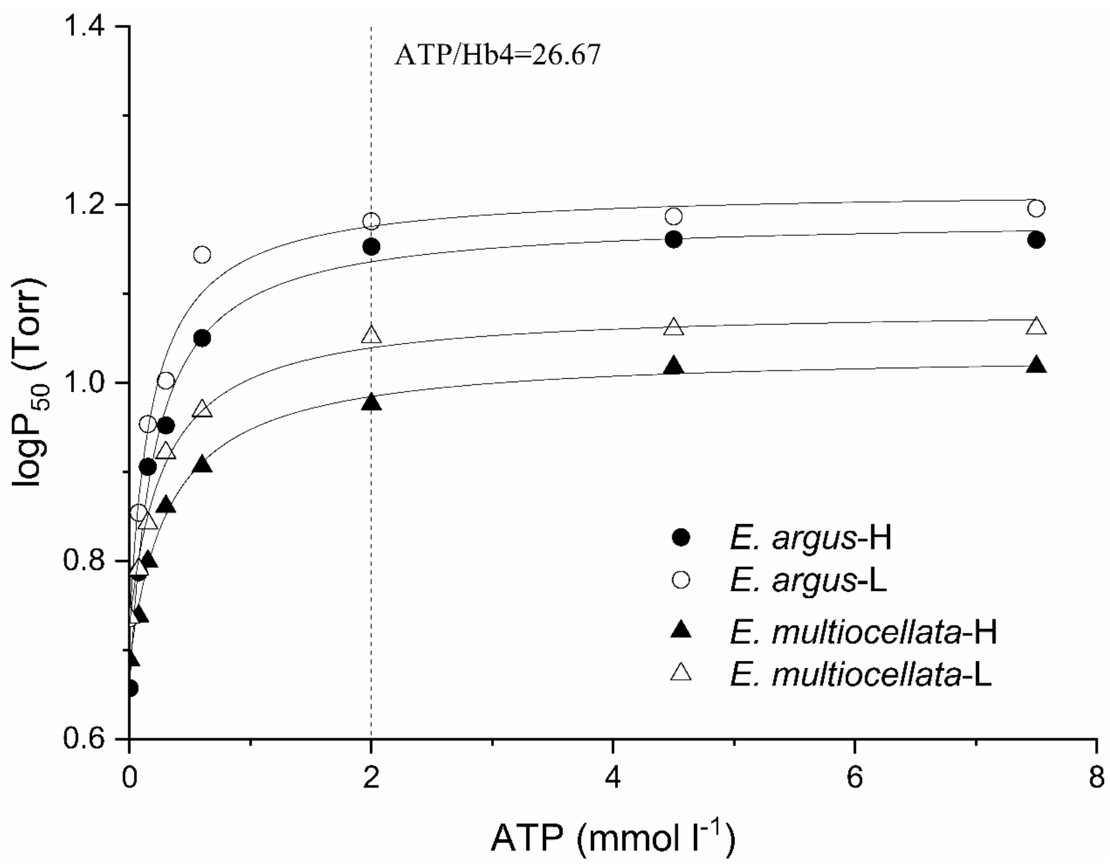

Figure S3. Dose-response curves showing effects of ATP on P_{50} of high- (solid) and low- (open) altitude *E. argus* (circle) and *E. multiocellata* (triangle) Hbs measured at 37 °C pH7.4 in the absence of Cl^-. n = 6 lizards for each population and 6 technical repeats were performed for each experiment condition.

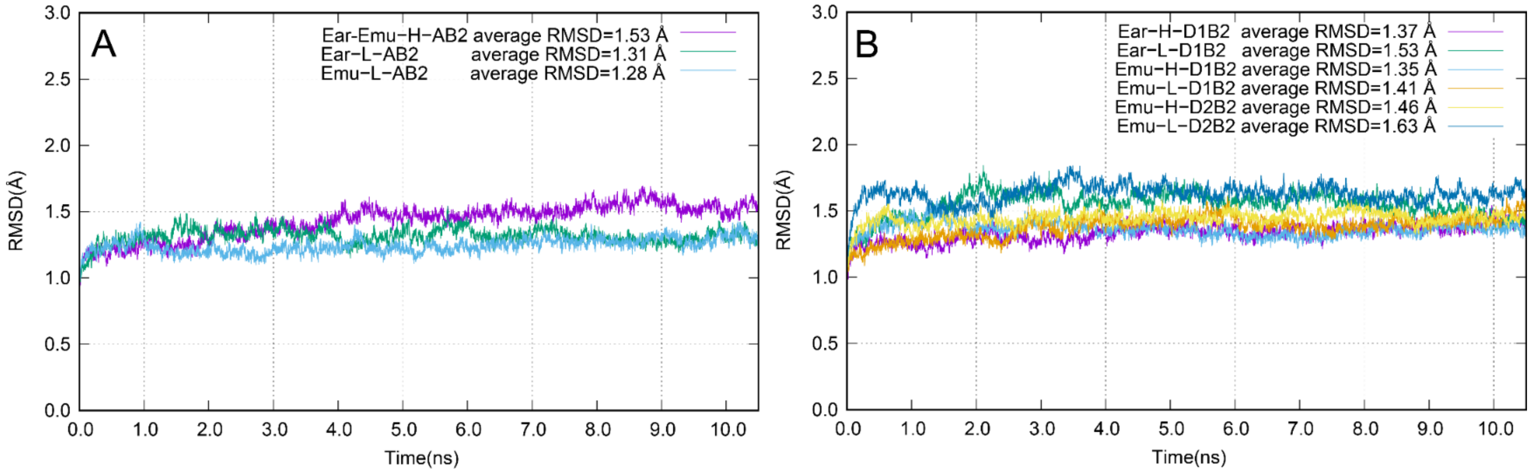

Figure S4 Evolution of root mean square deviation (RMSD) of the atoms in the backbone of the Hbs over time in AB2 Hb models (A) molecular dynamics simulations and DB2 Hb models (B) molecular dynamics simulations. The average RMSD over the last 4 ns for AB2 and DB2 models were also shown in A and B. Ear-H and Ear-L indicate the high- and low-altitude *E. argus*, Emu-H, and Emu-L indicate the high- and low-altitude *E. multiocellata.* AB2, D1B2 and D2B2 indicate the Hb models formed by αA, αD1, αD2 and β2 globin, respectively. Ear-Emu-H-AB2 indicates the same AB2 (αAβ2) Hb model of high-altitude *E. argus* and *E. multiocellata* as their αA and β2 globins are the same.
